# Sharp bounds for the Sándor–Yang means in terms of arithmetic and contra-harmonic means

**DOI:** 10.1186/s13660-018-1719-6

**Published:** 2018-05-30

**Authors:** Hui-Zuo Xu, Yu-Ming Chu, Wei-Mao Qian

**Affiliations:** 1 School of Economics and Management, Wenzhou Broadcast and TV University, Wenzhou, China; 2 Department of Mathematics, Huzhou University, Huzhou, China; 3 School of Distance Education, Huzhou Broadcast and TV University, Huzhou, China

**Keywords:** 26E60, 26D07, 26D99, Schwab–Borchardt mean, Sándor–Yang mean, Arithmetic mean, Contra-harmonic mean, Quadratic mean

## Abstract

In the article, we provide several sharp upper and lower bounds for two Sándor–Yang means in terms of combinations of arithmetic and contra-harmonic means.

## Preliminaries

Let $a, b>0$ with $a\neq b$. Then the arithmetic mean $A(a,b)$ [1–4], the quadratic mean $Q(a,b)$ [5], the contra-harmonic mean $C(a, b)$ [6–9], the Neuman–Sándor mean $NS(a, b)$ [10–12], the second Seiffert mean $T(a, b)$ [13, 14], and the Schwab–Borchardt mean $SB(a, b)$ [15, 16] of *a* and *b* are defined by

1.1 $$\begin{aligned} &A(a,b)=\frac{a+b}{2}, \qquad Q(a,b)=\sqrt{\frac{a^{2}+b^{2}}{2}}, \qquad C(a,b)=\frac{a^{2}+b^{2}}{a+b}, \end{aligned}$$

1.2 $$\begin{aligned} \ &NS(a,b)=\frac{a-b}{2\sinh^{-1} (\frac{a-b}{a+b} )}, \end{aligned}$$

1.3 $$\begin{aligned} &T(a,b)=\frac{a-b}{2\arctan (\frac{a-b}{a+b} )}, \\ &SB(a,b)=\textstyle\begin{cases} \frac{\sqrt{b^{2}-a^{2}}}{\arccos(a/b)}, & a< b,\\ \frac{\sqrt{a^{2}-b^{2}}}{\cosh^{-1}(a/b)}, & a>b, \end{cases}\displaystyle \end{aligned}$$

respectively, where $\sinh^{-1}(x)=\log(x+\sqrt{x^{2}+1})$ and $\cosh^{-1}(x)=\log(x+\sqrt{x^{2}-1})$ are respectively the inverse hyperbolic sine and cosine functions. The Schwab–Borchardt mean $SB(a,b)$ is strictly increasing, non-symmetric and homogeneous of degree one with respect to its variables. It can be expressed by the degenerated completely symmetric elliptic integral of the first kind [17]. Recently, the Schwab–Borchardt mean has attracted the attention of many researchers. In particular, many remarkable inequalities for the Schwab–Borchardt mean and its generated means can be found in the literature [18–38].

Let $X(a,b)$ and $Y(a,b)$ denote symmetric bivariate means of *a* and *b*. Then Yang [39] introduced the Sándor–Yang mean

$$ R_{XY}(a,b)=Y(a,b)e^{\frac{X(a,b)}{SB[X(a,b), Y(a,b)]}-1} $$

and presented the explicit formulas for $R_{QA}(a,b)$ and $R_{AQ}(a,b)$ as follows:

1.4 $$\begin{aligned} &R_{QA}(a,b)=A(a,b)e^{\frac{Q(a,b)}{NS(a,b)}-1}, \end{aligned}$$

1.5 $$\begin{aligned} &R_{AQ}(a,b)=Q(a,b)e^{\frac{A(a,b)}{T(a,b)}-1}. \end{aligned}$$

Very recently, the bounds involving the Sándor–Yang means have been the subject of intensive research. Numerous interesting results and inequalities for $R_{QA}(a,b)$ and $R_{AQ}(a,b)$ can be found in the literature [40–42].

Neuman [43] established the inequality

1.6 $$ R_{AQ}(a,b)< R_{QA}(a,b) $$

for $a,b > 0$ with $a\neq b$.

In [44], Xu proved that the double inequalities

1.7 $$ \begin{aligned} &\alpha_{1}C(a,b)+(1- \alpha_{1})A(a,b)< R_{QA}(a,b)< \beta_{1}C(a,b)+(1- \beta_{1})A(a,b), \\ &\alpha_{2}C(a,b)+(1-\alpha_{2})A(a,b)< R_{AQ}(a,b)< \beta_{2}C(a,b)+(1-\beta_{2})A(a,b) \end{aligned} $$

hold for all $a,b > 0$ with $a\neq b$ if and only if $\alpha_{1}\leq (1+\sqrt{2})^{\sqrt{2}}/e-1=0.2794\ldots$ , $\beta_{1}\geq 1/3$, $\alpha_{2}\leq \sqrt{2}e^{\pi/4-1}-1=0.1410\ldots$ and $\beta_{2}\geq 1/6$.

From (1.6) and (1.7), together the well-known inequalities

$$ C(a,b)> Q(a,b)> A(a,b), \qquad Q(a,b)>\frac{1}{3}C(a,b)+ \frac{2}{3}A(a,b), $$

we clearly see that

1.8 $$ A(a,b)< R_{AQ}(a,b)< R_{QA}(a,b)< Q(a,b)< C(a,b) $$

for all $a, b >0$ with $a\neq b$.

The main purpose of this paper is to find the best possible parameters $\alpha_{i}, \beta_{i}\in (0, 1)$ $(i=1, 2, 3, 4)$ such that the double inequalities

$$\begin{aligned} &C^{\alpha_{1}}(a,b)A^{1-\alpha_{1}}(a,b)< R_{QA}(a,b)< C^{\beta_{1}}(a,b)A^{1-\beta_{1}}(a,b), \\ &C^{\alpha_{2}}(a,b)A^{1-\alpha_{2}}(a,b)< R_{AQ}(a,b)< C^{\beta_{2}}(a,b)A^{1-\beta_{2}}(a,b), \\ &\alpha_{3} \biggl[\frac{1}{3}C(a,b)+\frac{2}{3}A(a,b) \biggr]+(1-\alpha_{3})C^{1/3}(a,b)A^{2/3}(a,b) \\ &\quad < R_{QA}(a,b)< \beta_{3} \biggl[\frac{1}{3}C(a,b)+ \frac{2}{3}A(a,b) \biggr]+(1-\beta_{3})C^{1/3}(a,b)A^{2/3}(a,b), \\ &\alpha_{4} \biggl[\frac{1}{6}C(a,b)+\frac{5}{6}A(a,b) \biggr]+(1-\alpha_{4})C^{1/6}(a,b)A^{5/6}(a,b) \\ &\quad < R_{AQ}(a,b)< \beta_{4} \biggl[\frac{1}{6}C(a,b)+ \frac{5}{6}A(a,b) \biggr]+(1-\beta_{4})C^{1/6}(a,b)A^{5/6}(a,b) \end{aligned}$$

hold for all $a, b>0$ with $a\neq b$.

## Lemmas

In order to prove our main results, we need several lemmas, which we present in this section.

### Lemma 2.1

(see [45])

*Let* $a, b\in \mathbb{R}$ *with* $a< b$, $f, g: [a, b]\mapsto \mathbb{R}$ *be continuous on* $[a, b]$ *and differentiable on* $(a, b)$, *and* $g^{\prime}(x)\neq 0$ *on* $(a, b)$. *If* $f^{\prime}(x)/g^{\prime}(x)$ *is increasing* (*decreasing*) *on* $(a, b)$, *then so are the functions*

$$ \frac{f(x)-f(a)}{g(x)-g(a)}, \qquad \frac{f(x)-f(b)}{g(x)-g(b)}. $$

*If* $f^{\prime}(x)/g^{\prime}(x)$ *is strictly monotone*, *then the monotonicity in the conclusion is also strict*.

### Lemma 2.2

(see [46])

*Let* $A(t)=\sum_{k=0}^{\infty}a_{k}t^{k}$ *and* $B(t)=\sum_{k=0}^{\infty }b_{k}t^{k}$ *be two real power series converging on* $(-r,r)$ ($r>0$) *with* $b_{k}>0$ *for all* *k*. *If the non*-*constant sequence* $\{a_{k}/b_{k}\}_{k=0}^{\infty}$ *is increasing* (*decreasing*) *for all* *k*, *then the function* $t\mapsto A(t)/B(t)$ *is strictly increasing* (*decreasing*) *on* $(0,r)$.

### Lemma 2.3

*The function*

$$ \phi(x)=\frac{x\coth(x)-1}{2\log[\cosh(x)]} $$

*is strictly increasing from* $(0, \log(1+\sqrt{2})$ *onto* $(1/3, [\sqrt{2}\log(1+\sqrt{2})-1]/\log 2)$.

### Proof

Let ${\phi _{1}} ( x ) = x\coth ( x ) - 1$, ${\phi _{2}} ( x ) = 2\log [ {\cosh ( x )} ]$. Then elaborate computations lead to

2.1 $$\begin{aligned} &\phi ( x ) = \frac{{{\phi _{1}} ( x )}}{{{\phi _{2}} ( x )}} = \frac{{{\phi _{1}} ( x ) - {\phi _{1}} ( {{0^{+} }} )}}{{{\phi _{2}} ( x ) - {\phi _{2}} ( 0 )}}, \end{aligned}$$

2.2 $$\begin{aligned} &\begin{aligned}[b] \frac{{{{\phi }'_{1}} ( x )}}{{{{\phi }'_{2}} ( x )}} &= \frac{{\sinh ( x ){{\cosh }^{2}} ( x ) - x\cosh ( x )}}{{2{{\sinh }^{3}} ( x )}} \\ &= \frac{{\sinh ( {3x} ) + \sinh ( x ) - 4x\cosh ( x )}}{{2\sinh ( {3x} ) - 6\sinh ( x )}} = \frac{{\sum_{n = 0}^{\infty}{\frac{{{3^{2n + 1}} - 8n - 3}}{{ ( {2n + 1} )!}}{x^{2n + 1}}} }}{{\sum_{n = 0}^{\infty}{\frac{{6 ( {{3^{2n}} - 1} )}}{{ ( {2n + 1} )!}}{x^{2n + 1}}} }} \\ &= \frac{{\sum_{n = 1}^{\infty}{\frac{{{3^{2n + 1}} - 8n - 3}}{{ ( {2n + 1} )!}}{x^{2n + 1}}} }}{{\sum_{n = 1}^{\infty}{\frac{{6 ( {{3^{2n}} - 1} )}}{{ ( {2n + 1} )!}}{x^{2n + 1}}} }} = \frac{{\sum_{n = 0}^{\infty}{\frac{{{3^{2n + 3}} - 8n - 11}}{{ ( {2n + 3} )!}}{x^{2n + 3}}} }}{{\sum_{n = 0}^{\infty}{\frac{{6 ( {{3^{2n{\mathrm{ + }}2}} - 1} )}}{{ ( {2n + 3} )!}}{x^{2n + 3}}} }}. \end{aligned} \end{aligned}$$

Let

2.3 $$ {a_{n}} = \frac{{{3^{2n + 3}} - 8n - 11}}{{ ( {2n + 3} )!}}, \qquad {b_{n}} = \frac{{6 ( {{3^{2n{\mathrm{ + }}2}} - 1} )}}{{ ( {2n + 3} )!}}. $$

Then

2.4 $$ {b_{n}} > 0 $$

and

2.5 $$ \frac{{{a_{n + 1}}}}{{{b_{n + 1}}}} - \frac{{{a_{n}}}}{{{b_{n}}}} = \frac{{4 [ { ( {72n + 63} ){3^{2n}} + 1} ]}}{ {3 ( {{3^{2n + 4}} - 1} ) ( {{3^{2n{\mathrm{ + }}2}} - 1} )}} > 0 $$

for all $n \ge 0$.

It follows from Lemma 2.2 and (2.2)–(2.5) that ${\phi '_{1}} ( x )/{\phi '_{2}} ( x )$ is strictly increasing on $( {0,\log ( {1 + \sqrt {2} } )} )$.

Note that

2.6 $$ \phi \bigl( {{0^{+} }} \bigr) = \frac{{{a_{0}}}}{{{b_{0}}}} = \frac{1}{3}, \qquad \phi \bigl( {\log ( {1 + \sqrt {2} } )} \bigr) = \frac{{\sqrt {2} \log ( {1 + \sqrt {2} } ) - 1}}{{\log 2}} = 0.3555 \ldots. $$

Therefore, Lemma 2.3 follows from Lemma 2.1, (2.1), and (2.6) together with the monotonicity of ${\phi '_{1}} ( x )/{\phi '_{2}} ( x )$. □

### Lemma 2.4

*The function*

$$ \varphi(x)=\frac{\log\sec(x)+x\cot(x)-1}{2\log\sec(x)} $$

*is strictly increasing from* $(0, \pi/4)$ *onto* $(1/6, 1/2-(4-\pi)(4\log 2))$.

### Proof

Let ${\varphi _{1}} ( x ) = \log \sec ( x ) + x\cot ( x ) - 1$, ${\varphi _{2}} ( x ) = 2\log [ {\sec ( x )} ]$, ${\varphi _{3}} ( x ) = \sin ( x ) - x\cos ( x )$, and ${\varphi _{4}} ( x ) = 2{\sin ^{3}} ( x )$. Then elaborate computations lead to

2.7 $$\begin{aligned} &\varphi ( x ) = \frac{{{\varphi _{1}} ( x )}}{{{\varphi _{2}} ( x )}} = \frac{{{\varphi _{1}} ( x ) - {\varphi _{1}} ( {{0^{+} }} )}}{{{\varphi _{2}} ( x ) - {\varphi _{2}} ( 0 )}}, \end{aligned}$$

2.8 $$\begin{aligned} &\frac{{{{\varphi }'_{1}} ( x )}}{{{{\varphi }'_{2}} ( x )}} = \frac{{{\varphi _{3}} ( x )}}{{{\varphi _{4}} ( x )}} = \frac{{{\varphi _{3}} ( x ) - {\varphi _{3}} ( 0 )}}{{{\varphi _{4}} ( x ) - {\varphi _{4}} ( 0 )}} \end{aligned}$$

and

2.9 $$ \frac{{{{\varphi }'_{3}} ( x )}}{{{{\varphi }'_{4}} ( x )}} = \frac{x}{{3\sin ( {2x} )}} = \frac{1}{6} \times \frac{1}{{\sin (2x)/ ( {2x} )}}. $$

It is well known that the function $x \to \sin ( x )/x$ is strictly decreasing on $( {0,\pi /2} )$, hence equation (2.9) leads to the conclusion that the function ${\varphi '_{3}} ( x )/{\varphi '_{4}} ( x )$ is strictly increasing on $( {0,\pi /4} )$.

Note that

2.10 $$ \begin{aligned} &\varphi \bigl( {{0^{+} }} \bigr) = \lim_{x \to {0^{+} }} \frac{{{{\varphi }'_{3}} ( x )}}{{{{\varphi }'_{4}} ( x )}} = \frac{1}{6}, \\ &\varphi \biggl( { \frac{\pi }{4}} \biggr) = \frac{1}{2} - \frac{{4 - \pi }}{{4\log 2}} = 0.1903 \dots. \end{aligned} $$

Therefore, Lemma 2.4 follows from Lemma 2.1 and (2.7)–(2.9) together with the monotonicity of ${\varphi '_{3}} ( x )/{\varphi '_{4}} ( x )$. □

### Lemma 2.5

*Let* $p \in ( {0,1} )$ *and*

$$ f ( x ) = 3{p^{2}} {x^{10}} + 14p ( {1 - p} ){x^{6}} + 18{p^{2}} {x^{4}} - 9{ ( {1 - p} )^{2}} {x^{2}} - 2p ( {1 - p} ). $$

*Then the following statements are true*:

1. *If* $p = 3/10$, *then* $f ( x ) > 0$ *for all* $x \in ( {1,\sqrt[6]{2}} )$;
2. *If* $p = 3 [ {{{ ( {1 + \sqrt {2} } )}^{\sqrt {2} }}/e - \sqrt[3]{2}} ]/ ( {4 - 3\sqrt[3]{2}} ) = 0.2663 \dots$ , *then there exists* ${\lambda _{0}}( = 1.0808 \dots ) \in ( {1,\sqrt[6]{2}} )$ *such that* $f ( x ) < 0$ *for* $x \in ( {1,{\lambda _{0}}} )$ *and* $f ( x ) > 0$ *for* $x \in ( {{\lambda _{0}},\sqrt[6]{2}} )$.

### Proof

Part $(1)$ follows easily from

$$ f ( x ) = \frac{3}{{100}} \bigl( {{x^{2}} - 1} \bigr) \bigl( {9{x^{8}} + 9{x^{6}} + 107{x^{4}} + 161{x^{2}} + 14} \bigr) > 0 $$

for all $x \in ( {1,\sqrt[6]{2}} )$ if $p = 3/10$.

For part $(2)$, if $p = 3 [ {{{ ( {1 + \sqrt {2} } )}^{\sqrt {2} }}/e - \sqrt[3]{2}} ]/ ( {4 - 3\sqrt[3]{2}} )$, then numerical computations lead to

2.11 $$\begin{aligned} &20p - 3 = 2.3273 \dots > 0, \end{aligned}$$

2.12 $$\begin{aligned} &f ( 1 ) = 3 ( {10p - 3} ) = - 1.008 \dots < 0, \end{aligned}$$

2.13 $$\begin{aligned} &f \bigl( {\sqrt[6]{2}} \bigr) = 1.6809 \dots > 0, \end{aligned}$$

2.14 $$\begin{aligned} &f' ( x ) = 30{p^{2}} {x^{9}} + 84p ( {1 - p} ){x^{5}} + 72{p^{2}} {x^{3}} - 18{ ( {1 - p} )^{2}}x. \end{aligned}$$

It follows from (2.11) and (2.14) that

2.15 $$ f' ( x ) > \bigl[ {30{p^{2}} + 84p ( {1 - p} ) + 72{p^{2}} - 18{{ ( {1 - p} )}^{2}}} \bigr]x = 6 ( {20p - 3} )x > 0 $$

for all $x \in ( {1,\sqrt[6]{2}} )$.

Therefore, part $(2)$ follows easily from (2.12), (2.13), (2.15), and the numerical results $f ( {1.0808} ) < 0$ and $f ( {1.0809} ) > 0$. □

### Lemma 2.6

*Let* $p \in ( {0,1} )$ *and*

$$ g ( x ) = 3{p^{2}} {x^{11}} + 56p ( {1 - p} ){x^{6}} + 75{p^{2}} {x^{5}} - 72{ ( {1 - p} )^{2}}x - 50p ( {1 - p} ). $$

*Then the following statements are true*:

1. *If* $p = 12/25$, *then* $g ( x ) > 0$ *for all* $x \in ( {1,\sqrt[6]{2}} )$;
2. *If* $p = 6 [ {\sqrt {2} {e^{\pi/4- 1}} - \sqrt[6]{2}} ]/ ( {7 - 6\sqrt[6]{2}} ) = 0.4210 \dots$ , *then there exists* ${\mu _{0}}( = 1.0577 \dots ) \in ( {1,\sqrt[6]{2}} )$ *such that* $g ( x ) < 0$ *for* $x \in ( {1,{\mu _{0}}} )$ *and* $g ( x ) > 0$ *for* $x \in ( {{\mu _{0}},\sqrt[6]{2}} )$.

### Proof

Part $(1)$ follows easily from

$$\begin{aligned} g ( x ) =& \frac{{24}}{{625}} ( {x - 1} ) \bigl( 18{x^{10}} + 18{x^{9}} + 18{x^{8}} + 18{x^{7}} + 18{x^{6}} + 382{x^{5}} + 832{x^{4}} \\ &{} + 832{x^{3}} + 832{x^{2}} + 832x + 325 \bigr) > 0 \end{aligned}$$

for all $x \in ( {1,\sqrt[6]{2}} )$ if $p = 12/25$.

For part $(2)$, if $p =6[\sqrt{2}e^{\pi/4-1}-\sqrt[6]{2}]/(7-6\sqrt[6]{2}) = 0.4210 \ldots$ , then numerical computations lead to

2.16 $$\begin{aligned} &20p - 3 = 5.4217 \dots > 0, \end{aligned}$$

2.17 $$\begin{aligned} &g ( 1 ) = 6 ( {25p - 12} ) = - 8.8367 \dots < 0, \end{aligned}$$

2.18 $$\begin{aligned} &g \bigl( {\sqrt[6]{2}} \bigr) = 13.6200 \dots > 0, \end{aligned}$$

2.19 $$\begin{aligned} &g' ( x ) = 3 \bigl[ {11{p^{2}} {x^{10}} + 112p ( {1 - p} ){x^{5}} + 125{p^{2}} {x^{4}} - 24{{ ( {1 - p} )}^{2}}} \bigr]. \end{aligned}$$

It follows from (2.16) and (2.19) that

2.20 $$\begin{aligned} g' ( x ) &> 11{p^{2}} + 112p ( {1 - p} ) + 125{p^{2}} - 24{ ( {1 - p} )^{2}} \\ &= 24 ( {20p - 3} ) > 0 \end{aligned}$$

for $x \in ( {1,\sqrt[6]{2}} )$.

Therefore, part $(2)$ follows easily from (2.17), (2.18), and (2.20) together with the numerical results $g ( {1.0577} ) < 0$ and $g ( {1.0578} ) > 0$. □

## Main results

We are now in a position to state and prove our main results.

### Theorem 3.1

*The double inequality*

3.1 $$ {C^{{\alpha _{1}}}} ( {a,b} ){A^{1 - {\alpha _{1}}}} ( {a,b} ) < {R_{QA}} ( {a,b} ) < {C^{{\beta _{1}}}} ( {a,b} ){A^{1 - {\beta _{1}}}} ( {a,b} ) $$

*holds for all* $a,b>0 $ *with* $a\ne b $ *if and only if* ${\alpha _{1}} \le 1/3 $ *and* ${\beta _{1}} \ge [ {\sqrt {2} \log ( {1 + \sqrt {2} } ) - 1} ]/\log 2 $.

### Proof

Clearly, inequality (3.1) can be rewritten as

3.2 $$ { \biggl[ {\frac{{C(a,b)}}{{A(a,b)}}} \biggr]^{{\alpha _{1}}}} < \frac{{{R_{QA}} ( {a,b} )}}{{A(a,b)}} < { \biggl[ {\frac{{C(a,b)}}{{A(a,b)}}} \biggr]^{{\beta _{1}}}}. $$

Since $A(a,b)$, ${R_{QA}} ( {a,b} )$, and $C(a,b)$ are symmetric and homogenous of degree one, we assume that $a > b > 0$. Let $v = ( {a - b} )/ ( {a + b} ) \in ( {0,1} )$. Then from (1.1), (1.2), and (1.4) we know that inequality (3.2) is equivalent to

3.3 $$ {\alpha _{1}} < \frac{{ [ {\sqrt {1 + {v^{2}}}\sinh^{-1} ( v )} ]/v - 1}}{{\log ( {1 + {v^{2}}} )}} < {\beta _{1}}. $$

Let $x =\sinh^{-1} ( v )$. Then $x \in ( {0,\log ( {1 + \sqrt {2} } )} )$ and

3.4 $$ \frac{{ [ {\sqrt {1 + {v^{2}}}\sinh^{-1} ( v )} ]/v - 1}}{{\log ( {1 + {v^{2}}} )}} = \frac{{x\coth ( x ) - 1}}{{2\log [ {\cosh ( x )} ]}}: = \phi ( x ). $$

Therefore, inequality (3.1) holds for all $a,b > 0$ with $a \ne b$ if and only if ${\alpha _{1}} \le 1/3$ and ${\beta _{1}} \ge [ {\sqrt {2} \log ( {1 + \sqrt {2} } ) - 1} ]/\log 2$ follows from (3.2)–(3.4) and Lemma 2.3. □

### Theorem 3.2

*The double inequality*

3.5 $$ {C^{{\alpha _{2}}}} ( {a,b} ){A^{1 - {\alpha _{2}}}} ( {a,b} ) < {R_{AQ}} ( {a,b} ) < {C^{{\beta _{2}}}} ( {a,b} ){A^{1 - {\beta _{2}}}} ( {a,b} ) $$

*holds for all* $a,b>0 $ *with* $a\ne b $ *if and only if* ${\alpha _{2}} \le 1/6 $ *and* ${\beta _{2}} \ge 1/2 - ( {4 - \pi } )/ ( {4\log 2} ) = 0.1903 \dots $ .

### Proof

Clearly, inequality (3.5) can be rewritten as

3.6 $$ \biggl[ {\frac{{C(a,b)}}{{A(a,b)}}} \biggr]^{{\alpha _{2}}} < \frac{{{R_{AQ}} ( {a,b} )}}{{A(a,b)}} < { \biggl[ {\frac{{C(a,b)}}{{A(a,b)}}} \biggr]^{{\beta _{2}}}}. $$

Since $A(a,b)$, ${R_{AQ}} ( {a,b} )$, and $C(a,b)$ are symmetric and homogenous of degree one, we assume that $a > b > 0$. Let $v = ( {a - b} )/ ( {a + b} ) \in ( {0,1} )$. Then from (1.1), (1.3), and (1.5) we see that inequality (3.6) is equivalent to

3.7 $$ {\alpha _{2}} < \frac{{\log \sqrt {1 + {v^{2}}} + [ {\arctan ( v )} ]/v - 1}}{{\log ( {1 + {v^{2}}} )}} < {\beta _{2}}. $$

Let $x = \arctan ( v )$. Then $x \in ( {0,\pi /4} ) $ and

3.8 $$\begin{aligned} &\frac{{\log \sqrt {1 + {v^{2}}} + [ {\arctan ( v )} ]/v - 1}}{{\log ( {1 + {v^{2}}} )}} \\ &\quad = \frac{{\log \sec ( x ) + x\cot ( x ) - 1}}{{2\log \sec ( x )}}: = \varphi ( x ). \end{aligned}$$

Therefore, inequality (3.5) holds for all $a,b > 0$ with $a \ne b$ if and only if ${\alpha _{2}} \le 1/6 $ and ${\beta _{2}} \ge 1/2 - ( {4 - \pi } )/ ( {4\log 2} ) = 0.1903 \dots $ follows from (3.6)–(3.8) and Lemma 2.4. □

### Theorem 3.3

*The double inequality*

$$\begin{aligned} &{\alpha _{3}} \biggl[ {\frac{1}{3}C ( {a,b} ) + \frac{2}{3}A ( {a,b} )} \biggr] + ( {1 - {\alpha _{3}}} ){C^{1/3}} ( {a,b} ){A^{2/3}} ( {a,b} ) \\ &\quad < {R_{QA}} ( {a,b} ) < {\beta _{3}} \biggl[ { \frac{1}{3}C ( {a,b} ) + \frac{2}{3}A ( {a,b} )} \biggr] + ( {1 - { \beta _{3}}} ){C^{1/3}} ( {a,b} ){A^{2/3}} ( {a,b} ) \end{aligned}$$

*holds for all* $a,b>0 $ *with* $a\ne b $ *if and only if* ${\alpha _{3}} \le 3 [ {{{ ( {1 + \sqrt {2} } )}^{\sqrt {2} }}/e - \sqrt[3]{2}} ]/ ( {4 - 3\sqrt[3]{2}} ) = 0.2663 \dots $ *and* ${\beta _{3}} \ge 3/10 $.

### Proof

Since ${R_{QA}} ( {a,b} )$, $A(a,b)$, and $C(a,b)$ are symmetric and homogenous of degree one, without loss generality, we assume that $a > b > 0$. Let $v = ( {a - b} )/ ( {a + b} ) $, $x = \sqrt[6]{{1 + {v^{2}}}}$, and $p \in ( {0,1} )$. Then $v \in ( {0,1} )$, $x \in ( {1,\sqrt[6]{2}} ) $, and (1.1), (1.2), and (1.4) lead to

3.9 $$\begin{aligned} &\log \frac{{{R_{QA}} ( {a,b} )}}{{p [ {\frac{1}{3}C ( {a,b} ) + \frac{2}{3}A ( {a,b} )} ] + ( {1 - p} ){C^{1/3}} ( {a,b} ){A^{2/3}} ( {a,b} )}} \\ &\quad = \frac{{\sqrt {1 + {v^{2}}} \sinh^{-1} ( v )}}{v} - \log \biggl[ {p \biggl( {\frac{1}{3}{v^{2}} + 1} \biggr) + ( {1 - p} )\sqrt[3]{{1 + {v^{2}}}}} \biggr] - 1 \\ &\quad = \frac{{{x^{3}}\sinh^{-1} ( {\sqrt {{x^{6}} - 1} } )}}{{\sqrt {{x^{6}} - 1} }} - \log \biggl[ {p \biggl( {\frac{1}{3}{x^{6}} + \frac{2}{3}} \biggr) + ( {1 - p} ){x^{2}}} \biggr] - 1. \end{aligned}$$

Let

3.10 $$ F ( x ) = \frac{{{x^{3}}\sinh^{-1} ( {\sqrt {{x^{6}} - 1} } )}}{{\sqrt {{x^{6}} - 1} }} - \log \biggl[ {p \biggl( { \frac{1}{3}{x^{6}} + \frac{2}{3}} \biggr) + ( {1 - p} ){x^{2}}} \biggr] - 1. $$

Then simple computations lead to

3.11 $$\begin{aligned} &F \bigl( {{1^{+} }} \bigr) = 0, \qquad F \bigl( {\sqrt[6]{2}} \bigr) = \sqrt {2} \log ( {1 + \sqrt {2} } ) - \log \biggl[ {\frac{4}{3}p + \sqrt[3]{2} ( {1 - p} )} \biggr] - 1, \end{aligned}$$

3.12 $$\begin{aligned} &F' ( x ) = \frac{{3{x^{2}}}}{{{{ ( {{x^{6}} - 1} )}^{3/2}}}}{F_{1}} ( x ), \end{aligned}$$

where

3.13 $$\begin{aligned} &{F_{1}} ( x ) = \frac{{\sqrt {{x^{6}} - 1} [ { - p{x^{10}} + ( {1 - p} ){x^{6}} + 4p{x^{4}} + 2 ( {1 - p} )} ]}}{{x [ {p ( {{x^{6}} + 2} ) + 3 ( {1 - p} ){x^{2}}} ]}} - \sinh^{-1} \bigl( { \sqrt {{x^{6}} - 1} } \bigr), \\ &{F_{1}} ( 1 ) = 0, \qquad {F_{1}} \bigl( { \sqrt[6]{2}} \bigr) = \frac{{ ( {\sqrt[6]{{2048}} - 2\sqrt {2} } )p - \sqrt[6]{{2048}}}}{{3\sqrt[3]{2}p - 3\sqrt[3]{2} - 4p}} - \log ( {1 + \sqrt {2} } ), \end{aligned}$$

3.14 $$\begin{aligned} &{F'_{1}} ( x ) = - \frac{{2{{ ( {{x^{6}} - 1} )}^{3/2}}}}{{{x^{2}}{{ [ {p ( {{x^{6}} + 2} ) + 3 ( {1 - p} ){x^{2}}} ]}^{2}}}}f ( x ), \end{aligned}$$

where $f ( x ) $ is defined as in Lemma 2.5.

We divide the proof into four cases.

*Case 1* $p = 3/10 $. Then it follows from (3.9)–(3.14) and Lemma 2.5(1) that

$$ {R_{QA}} ( {a,b} ) < \frac{3}{{10}} \biggl[ {\frac{1}{3}C ( {a,b} ) + \frac{2}{3}A ( {a,b} )} \biggr] + \frac{7}{{10}}{C^{1/3}} ( {a,b} ){A^{2/3}} ( {a,b} ). $$

*Case 2* $0 < p < 3/10$. Let $v > 0 $ and $v \to {0^{+} } $. Then power series expansion leads to

3.15 $$\begin{aligned} &\frac{{\sqrt {1 + {v^{2}}} \sinh^{-1} ( v )}}{v} - \log \biggl[ {p \biggl( { \frac{1}{3}{v^{2}} + 1} \biggr) + ( {1 - p} )\sqrt[3]{{1 + {v^{2}}}}} \biggr] - 1 \\ &\quad = \biggl( {\frac{1}{{30}} - \frac{1}{9}p} \biggr){v^{4}} + O \bigl( {{v^{6}}} \bigr). \end{aligned}$$

Equations (3.9), (3.10), and (3.15) lead to the conclusion that there exists $0 < {\delta _{1}} < 1$ such that

$$ {R_{QA}} ( {a,b} ) > p \biggl[ {\frac{1}{3}C ( {a,b} ) + \frac{2}{3}A ( {a,b} )} \biggr] + ( {1 - p} ){C^{1/3}} ( {a,b} ){A^{2/3}} ( {a,b} ) $$

for all $a > b > 0$ with $( {a - b} )/ ( {a + b} ) \in ( {0,{\delta _{1}}} )$.

*Case 3* $p = 3 [ {{{ ( {1 + \sqrt {2} } )}^{\sqrt {2} }}/e - \sqrt[3]{2}} ]/ ( {4 - 3\sqrt[3]{2}} )$. Then (3.13) leads to

3.16 $$ {F_{1}} \bigl( {\sqrt[6]{2}} \bigr) = - 0.0039 \dots < 0. $$

Let ${\lambda _{0}} = 1.0808 \dots $ be the number given in Lemma 2.5(2). Then we divide the discussion into two subcases.

*Subcase 1* $x \in ( {1,{\lambda _{0}}} ]$. Then ${F_{1}} ( x ) > 0$ for $x \in ( {1,{\lambda _{0}}} ]$ follows easily from (3.13) and (3.14) together with Lemma 2.5(2).

*Subcase 2* $x \in ( {{\lambda _{0}},\sqrt[6]{2}} )$. Then Lemma 2.5(2) and (3.14) lead to the conclusion that ${F_{1}} ( x )$ is strictly decreasing on the interval $[ {{\lambda _{0}},\sqrt[6]{2}} )$. Then, from (3.16) and Subcase 1, we know that there exists ${\lambda _{1}} \in ( {{\lambda _{0}},\sqrt[6]{2}} )$ such that ${F_{1}} ( x ) > 0$ for $x \in [ {{\lambda _{0}},{\lambda _{1}}} )$ and ${F_{1}} ( x ) < 0$ for $x \in ( {{\lambda _{1}},\sqrt[6]{2}} )$.

It follows from Subcases 1 and 2 together with (3.12) that $F ( x )$ is strictly increasing on $( {1,{\lambda _{1}}} ]$ and strictly decreasing on $[ {{\lambda _{1}},\sqrt[6]{2}} )$. Therefore,

$$ {R_{QA}} ( {a,b} ) > p \biggl[ {\frac{1}{3}C ( {a,b} ) + \frac{2}{3}A ( {a,b} )} \biggr] + ( {1 - p} ){C^{1/3}} ( {a,b} ){A^{2/3}} ( {a,b} ) $$

follows from (3.9)–(3.11) and (3.16) together with the piecewise monotonicity of $F ( x )$.

*Case 4* $3 [ {{{ ( {1 + \sqrt {2} } )}^{\sqrt {2} }}/e - \sqrt[3]{2}} ]/ ( {4 - 3\sqrt[3]{2}} ) < p < 1$. Then (3.11) leads to

3.17 $$ F \bigl( {\sqrt[6]{2}} \bigr) = \sqrt {2} \log ( {1 + \sqrt {2} } ) - \log \biggl[ {\frac{4}{3}p + \sqrt[3]{2} ( {1 - p} )} \biggr] - 1 < 0. $$

Equations (3.9) and (3.10) together with inequality (3.17) imply that there exists $0 <\delta_{1}^{\ast} < 1$ such that

$$ {R_{QA}} ( {a,b} ) < p \biggl[ {\frac{1}{3}C ( {a,b} ) + \frac{2}{3}A ( {a,b} )} \biggr] + ( {1 - p} ){C^{1/3}} ( {a,b} ){A^{2/3}} ( {a,b} ) $$

for all $a > b > 0$ with $( {a - b} )/ ( {a + b} ) \in ( {1 -\delta_{1}^{\ast} ,1} )$. □

### Theorem 3.4

*The double inequality*

$$\begin{aligned} &{\alpha _{4}} \biggl[ {\frac{1}{6}C ( {a,b} ) + \frac{5}{6}A ( {a,b} )} \biggr] + ( {1 - {\alpha _{4}}} ){C^{1/6}} ( {a,b} ){A^{5/6}} ( {a,b} ) \\ &\quad< {R_{AQ}} ( {a,b} ) < {\beta _{4}} \biggl[ {\frac{1}{6}C ( {a,b} ) + \frac{5}{6}A ( {a,b} )} \biggr] + ( {1 - {\beta _{4}}} ){C^{1/6}} ( {a,b} ){A^{5/6}} ( {a,b} ) \end{aligned}$$

*holds for all* $a,b>0 $ *with* $a\ne b $ *if and only if* ${\alpha _{4}} \le 6 [ {\sqrt {2} {{\mathrm{e}} ^{ ( {\pi /4 - 1} )}} - \sqrt[6]{2}} ]/ ( {7 - 6\sqrt[6]{2}} ) = 0.4210 \dots $ *and* ${\beta _{4}} \ge 12/25 $.

### Proof

Since ${R_{AQ}} ( {a,b} )$, $A(a,b)$, and $C(a,b)$ are symmetric and homogenous of degree one, without loss generality, we assume that $a > b > 0$. Let $v = ( {a - b} )/ ( {a + b} ) $, $x = \sqrt[6]{{1 + {v^{2}}}}$, and $p \in ( {0,1} )$. Then $v \in ( {0,1} )$, $x \in ( {1,\sqrt[6]{2}} ) $ and (1.1), (1.3), and (1.5) lead to

3.18 $$\begin{aligned} &\log \frac{{{R_{AQ}} ( {a,b} )}}{{p [ {\frac{1}{6}C ( {a,b} ) + \frac{5}{6}A ( {a,b} )} ] + ( {1 - p} ){C^{1/6}} ( {a,b} ){A^{5/6}} ( {a,b} )}} \\ &\quad = \log \sqrt {1 + {v^{2}}} + \frac{{\arctan ( v )}}{v} - \log \biggl[ {p \biggl( {\frac{1}{6}{v^{2}} + 1} \biggr) + ( {1 - p} ) \sqrt[6]{{1 + {v^{2}}}}} \biggr] - 1 \\ &\quad = 3\log ( x ) + \frac{{\arctan ( {\sqrt {{x^{6}} - 1} } )}}{{\sqrt {{x^{6}} - 1} }} - \log \biggl[ {p \biggl( { \frac{1}{6}{x^{6}} + \frac{5}{6}} \biggr) + ( {1 - p} )x} \biggr] - 1. \end{aligned}$$

Let

3.19 $$ G ( x ) = 3\log ( x ) + \frac{{\arctan ( {\sqrt {{x^{6}} - 1} } )}}{{\sqrt {{x^{6}} - 1} }} - \log \biggl[ {p \biggl( {\frac{1}{6}{x^{6}} + \frac{5}{6}} \biggr) + ( {1 - p} )x} \biggr] - 1. $$

Then simple computations lead to

3.20 $$\begin{aligned} &G \bigl( {{1^{+} }} \bigr) = 0, \qquad G \bigl( {\sqrt[6]{2}} \bigr) = \log ( {\sqrt {2} } ) + \frac{\pi }{4} - \log \biggl[ { \frac{7}{6}p + \sqrt[6]{2} ( {1 - p} )} \biggr] - 1, \end{aligned}$$

3.21 $$\begin{aligned} &G' ( x ) = \frac{{3{x^{5}}}}{{{{ ( {{x^{6}} - 1} )}^{3/2}}}}{G_{1}} ( x ), \end{aligned}$$

where

3.22 $$\begin{aligned} &{G_{1}} ( x ) = \frac{{\sqrt {{x^{6}} - 1} [ { - p{x^{11}} + 4 ( {1 - p} ){x^{6}} + 7p{x^{5}} + 2 ( {1 - p} )} ]}}{{{x^{5}} [ {p ( {{x^{6}} + 5} ) + 6 ( {1 - p} )x} ]}} - \arctan \bigl( {\sqrt {{x^{6}} - 1} } \bigr), \\ &{G_{1}} ( 1 ) = 0, \qquad {G_{1}} \bigl( { \sqrt[6]{2}} \bigr) = \frac{{5 [ {\sqrt[6]{2} ( {1 - p} ) + p} ]}}{{6\sqrt[6]{2} ( {1 - p} ) + 7p}} - \frac{\pi }{4}, \end{aligned}$$

3.23 $$\begin{aligned} &{G'_{1}} ( x ) = - \frac{{{{ ( {{x^{6}} - 1} )}^{3/2}}}}{{{x^{6}}{{ [ {p ( {{x^{6}} + 5} ) + 6 ( {1 - p} )x} ]}^{2}}}}g ( x ), \end{aligned}$$

where $g ( x ) $ is defined as in Lemma 2.6.

We divide the proof into four cases.

*Case 1* $p = 12/25 $. Then it follows from (3.18)–(3.23) and Lemma 2.6(1) that

$$ {R_{AQ}} ( {a,b} ) < \frac{{12}}{{25}} \biggl[ {\frac{1}{3}C ( {a,b} ) + \frac{2}{3}A ( {a,b} )} \biggr] + \frac{{13}}{{25}}{C^{1/3}} ( {a,b} ){A^{2/3}} ( {a,b} ). $$

*Case 2* $0 < p < 12/25$. Let $v > 0 $ and $v \to {0^{+} } $, then power series expansion leads to

3.24 $$\begin{aligned} &\log \sqrt {1 + {v^{2}}} + \frac{{\arctan ( v )}}{v} - \log \biggl[ {p \biggl( {\frac{1}{6}{v^{2}} + 1} \biggr) + ( {1 - p} ) \sqrt[6]{{1 + {v^{2}}}}} \biggr] - 1 \\ &\quad = \biggl( {\frac{1}{{30}} - \frac{5}{{72}}p} \biggr){v^{4}} + O \bigl( {{v^{6}}} \bigr). \end{aligned}$$

Equations (3.18), (3.19), and (3.24) lead to the conclusion that there exists $0 < {\delta _{2}} < 1$ such that

$$ {R_{AQ}} ( {a,b} ) > p \biggl[ {\frac{1}{3}C ( {a,b} ) + \frac{2}{3}A ( {a,b} )} \biggr] + ( {1 - p} ){C^{1/3}} ( {a,b} ){A^{2/3}} ( {a,b} ) $$

for all $a > b > 0$ with $( {a - b} )/ ( {a + b} ) \in ( {0,{\delta _{2}}} )$.

*Case 3* $p = 6 [ {\sqrt {2} {{\mathrm{e}} ^{ ( {\pi /4 - 1} )}} - \sqrt[6]{2}} ]/ ( {7 - 6\sqrt[6]{2}} )$. Then, from (3.20) and (3.22) together with numerical computations, we get

3.25 $$ G \bigl( {\sqrt[6]{2}} \bigr) = 0, \qquad {G_{1}} \bigl( {\sqrt[6]{2}} \bigr) = - 0.0033 \dots < 0. $$

Let ${\mu _{0}} = 1.0577 \dots $ be the number given in Lemma 2.6(2). Then we divide the discussion into two subcases.

*Subcase 1* $x \in ( {1,{\mu _{0}}} ] $. Then ${G_{1}} ( x ) > 0$ for $x \in ( {1,{\mu _{0}}} ]$ follows easily from (3.22) and (3.23) together with Lemma 2.6(2).

*Subcase 2* $x \in ( {{\mu _{0}},\sqrt[6]{2}} )$. Then Lemma 2.6(2) and (3.23) lead to the conclusion that ${G_{1}} ( x )$ is strictly decreasing on the interval $[ {{\mu _{0}},\sqrt[6]{2}} )$. Then, from (3.25) and Subcase 1, we know that there exists ${\mu _{1}} \in ( {{\mu _{0}},\sqrt[6]{2}} )$ such that ${G_{1}} ( x ) > 0$ for $x \in [ {{\mu _{0}},{\mu _{1}}} )$ and ${G_{1}} ( x ) < 0$ for $x \in ( {{\mu _{1}},\sqrt[6]{2}} )$.

It follows from Subcases 1 and 2 together with (3.21) that $G ( x )$ is strictly increasing on $( {1,{\mu _{1}}} ]$ and strictly decreasing on $[ {{\mu _{1}},\sqrt[6]{2}} )$. Therefore,

$$ {R_{AQ}} ( {a,b} ) > p \biggl[ {\frac{1}{3}C ( {a,b} ) + \frac{2}{3}A ( {a,b} )} \biggr] + ( {1 - p} ){C^{1/3}} ( {a,b} ){A^{2/3}} ( {a,b} ) $$

follows from (3.18)–(3.20) and (3.25) together with the piecewise monotonicity of $G ( x )$.

*Case 4* $6 [ {\sqrt {2} {{\mathrm{e}} ^{ ( {\pi /4 - 1} )}} - \sqrt[6]{2}} ]/ ( {7 - 6\sqrt[6]{2}} ) < p < 1 $. Then (3.21) leads to

3.26 $$ G \bigl( {\sqrt[6]{2}} \bigr) = \log ( {\sqrt {2} } ) + \frac{\pi }{4} - \log \biggl[ {\frac{7}{6}p + \sqrt[6]{2} ( {1 - p} )} \biggr] - 1 < 0. $$

Equations (3.18) and (3.19) together with inequality (3.26) imply that there exists $0 < \delta_{2}^{\ast}< 1$ such that

$$ {R_{AQ}} ( {a,b} ) < p \biggl[ {\frac{1}{3}C ( {a,b} ) + \frac{2}{3}A ( {a,b} )} \biggr] + ( {1 - p} ){C^{1/3}} ( {a,b} ){A^{2/3}} ( {a,b} ) $$

for all $a > b > 0$ with $( {a - b} )/ ( {a + b} ) \in ( {1 - \delta_{2}^{\ast}, 1} )$. □

## Results and discussion

In this paper, we provide the optimal upper and lower bounds for the Sándor–Yang means $R_{QA}(a,b)$ and $R_{AQ}(a,b)$ in terms of combinations of the arithmetic mean $A(a,b)$ and the contra-harmonic mean $C(a,b)$. Our approach may have further applications in the theory of bivariate means.

## Conclusion

In the article, we find several best possible bounds for the Sándor–Yang means $R_{QA}(a,b)$ and $R_{AQ}(a,b)$. These results are improvements and refinements of the previous results.
